# Stay mindfully active during the coronavirus pandemic: a feasibility study of mHealth-delivered mindfulness yoga program for people with Parkinson’s disease

**DOI:** 10.1186/s12906-022-03519-y

**Published:** 2022-02-07

**Authors:** Jojo Yan Yan Kwok, Jung Jae Lee, Edmond Pui Hang Choi, Pui Hing Chau, Man Auyeung

**Affiliations:** 1 School of Nursing, Li Ka Shing Faculty of Medicine, The University of Hong Kong, Hong Kong 4/F, William MW Mong Block, 21 Sassoon Road, Pokfulam, Hong Kong SAR China; 2 Department of Medicine, Pamela Youde Nethersole Eastern Hospital, Chai Wan, Hong Kong SAR, China

**Keywords:** Parkinson’s disease, Rehabilitation, Quality of life, Physical exercise, Psychological support, Symptom management, mHealth, Yoga, Mindfulness, Home-based

## Abstract

**Importance:**

Patients with long-term neurological conditions, such as Parkinson’s disease (PD), are particularly vulnerable to the public health measures taken to combat the COVID-19 pandemic. The inaccessibility of center-based rehabilitation further aggravated their motor dysfunctions as well as mental distress, leading to exacerbation of motor and non-motor symptoms, high healthcare utilization and worsened health-related quality of life (HRQOL).

**Objective:**

This study aimed to evaluate the feasibility, safety, and preliminary effects of the mHealth-delivered home-based mindfulness yoga program on functional balance, motor symptoms, mental health and HRQOL in patients with PD.

**Design, setting and participants:**

This prospective, single-arm, non-randomized feasibility study adopted a sequential explanatory mixed-method design. Adults (aged ≥ 18) with a clinical diagnosis of idiopathic Parkinson’s disease (Hoehn and Yahr stage I to III) who were able to stand unaided and walk with or without an assistive device were enrolled via convenience sampling.

**Intervention:**

Home-based mindfulness yoga training were delivered via video-conferencing software (Zoom) in eight bi-weekly 90-min sessions.

**Main outcomes and measures:**

This current study measured functional balance, motor symptoms, perceived balance confidence, perceived freezing of gait symptoms, anxiety and depression, mindfulness and HRQOL using a tele-assessment approach at baseline and 1-week post-intervention. All participants were invited to attend qualitative individual interviews to explore their experience of using online mindfulness yoga program as a lifestyle intervention for PD rehabilitation.

**Results:**

Among the ten patients, 80% completed the program with an adherence rate of 98.4%. All participants were able to learn and practice mindfulness yoga following the eight bi-weekly online mindfulness yoga training sessions, without any significant adverse events. Tele-assessment of outcomes were feasible and uneventful. Qualitative feedback revealed participants had a high preference of using the tele-rehabilitation approach to stay mindful and being active, both physically and socially, while confronting the changes brought by COVID-19 pandemic.

**Conclusions and relevance:**

The mHealth-delivered home-based mindfulness yoga intervention was feasible, safe, and well-accepted among people with PD to relieve the burden brought by COVID-19 pandemic. Future studies should adopt a design with enhanced rigor, a comparison group, and enlarged sample size to evaluate the efficacy of the program in patients with long-term neurological conditions and/or physical impairments. We recommend a longer intervention duration of at least 8 weeks to enhance the psychophysiological effects.

## Background

Between 47%–80% of people with Parkinson’s disease (PD) experience freezing of gait (FOG) [1], which is associated with impaired balance and mobility, falls, fall-related physical injuries and fractures, functional restriction, increased institutionalization risk, and mortality [2, 3]. Clinical guidelines and previous studies recommend a complementary mind–body approach to PD rehabilitation [4], including yoga, Tai Chi, and dance on improving the motor symptoms and HRQOL of patients with PD [5–8]. However, most programs are delivered face-to-face in center-based settings, which are often inaccessible to many patients due to resource limitations, distance, disability, and financial concerns while posing potential health risks in the long run.

For people with PD, exercise is a vital component for maintaining balance, mobility and activities of daily living. With the public health and virus-control measures taken to combat the current coronavirus disease 2019 (COVID-19) pandemic, such as self-quarantine, social distancing, restriction on group gatherings, and community center and gym facility closures, patients with PD are particularly vulnerable due to the reduced accessibility of exercise-related facilities. Many patients spend increased time at home with reduced physical activity; this situation subsequently exacerbates deleterious health consequences, such as physical and functional decline, emotional distress, comorbid conditions, and worsened HRQOL [9–11]. Hence, additional effort should be expended to test and translate evidence- and center-based rehabilitation practices into home-based settings to address the challenges imposed by the common barriers to participation in center-based rehabilitation and any future infectious disease outbreaks.

Indeed, effective delivery and adherence to rehabilitation programs are of high priority in PD care. In recent years, advances in technologies have provided new avenues for medical and public health practice. For example, mobile devices, such as smartphones, tablets, and laptops (mHealth), have been utilized for chronic illness care. The adoption of mHealth technology offers a promising platform for efficient and accessible intervention delivery [12]. Several studies have demonstrated the efficacy of mHealth interventions for improving the overall HRQOL of patients with chronic illnesses, such as chronic pain, cancer, generalized anxiety disorder and cardiovascular diseases [13, 14]. However, mHealth-based interventions for improving motor symptoms, particularly balance, mobility, and FOG symptoms, as well as mental health and HRQOL, in patients with PD have been limited. The delivery of exercise programs by using a mHealth approach may be ideal for reaching and supporting patients with PD to improve their chronic illness care during infectious disease pandemics, relieve mental distress, and help them stay active at home. Therefore, we further tailored a mindfulness yoga programme, which was delivered interactively through an online platform for PD rehabilitation. This feasibility study aimed to examine the acceptability, safety and implementation of this mHealth-delivered home-based mindfulness yoga program. To our knowledge, this is the first feasibility study that solely used a telemedicine approach to deliver mindfulness yoga intervention to enhance the functional balance, motor symptoms, mental health and HRQOL of patients with PD.

## Methods

### Study design

This prospective, single-armed, non-randomized feasibility study adopted a sequential explanatory mixed-methods design to examine the acceptability, safety, and implementation of a mHealth-delivered home-based mindfulness yoga program for PD patients. The study was approved by the corresponding ethics committee (HKU/HA HKW IRB: UW 19–535) in accordance with the Declaration of Helsinki.

### Study participants

Using convenience sampling, we recruited potential participants through a regional neurology outpatient clinic in Hong Kong from May 2020 to June 2020. Inclusion criteria are: (i) participants who had a clinical diagnosis of idiopathic PD with a disease severity rating of stage I–III on the Hoehn and Yahr scale (rated on a scale of I–V with high numbers indicating severe disease), (ii) were older than 18 years, (iii) able to stand unaided for ≥ 10 min, (iv) able to walk with/without any assistive device for ≥ 100 m, (v) have access to the Internet and any mobile device, such as a smartphone, tablet, and laptop, (vi) were willing to download Zoom as the training platform, and (vii) able to give written consent. Exclusion criteria are: (i) those who had been regularly practicing instructor-led mind–body interventions ≥ once a week during the past 6 months, (ii) were currently participating in other behavioral/ pharmacological trials, (iii) had significant cognitive impairment as indicated by the Abbreviated Mental Test [15] score < 6 (sensitivity of 96% and specificity of 94%), (iv) and/or have other contraindication/severe comorbidities (e.g., severe hearing/vision impairment) that might limit their full participation in the study.

### Interventions

For 4 weeks, the mHealth-delivered mindfulness yoga program was delivered to participants with biweekly 90-min training sessions through an enhanced online platform via Zoom, a video conferencing software. All training sessions were group-based and delivered by an intervener using an interactive, real-time approach. The program was tailored on the basis of the research team’s previous experience in center-based mindfulness yoga intervention for PD [6, 7], which comprised the mindfulness practice of modified yoga sequences for PD and mindfulness meditation and breathing techniques. Each item was modified to target the functional impairments, such as turning, asymmetric body alignment, and facial stiffness, induced by PD motor symptoms. We encouraged all participants to perform daily 15-min self-practice of mindful walking to deepen the mindfulness experience specific to balance, mobility, and FOG symptoms. Mindful walking is type of moving meditation, which can be practiced anywhere without restriction. During mindful walking, the participants were instructed to walk and shifts their weight and balance slowly, with conscious awareness of the body sensations and thoughts whilst walking. The theme of each session reflected one of the eight mindfulness attitudes: non-judgement, patience, beginner’s mind, trust, non-striving, acceptance, letting go, and generosity [16]. The components of the mindfulness yoga program are presented in Table 1. At the end of each session, the intervener facilitated mindfulness practice and experiences to consolidate learning and promote social interaction. The major components, including the same set of yoga sequences, mindful walking, mindfulness meditation, and breathing techniques (http://yoga.nursing.hku.hk/), of the previously tested mindfulness yoga program were revamped into a series of guided videos and stored on a webpage for self-practice to promote skill mastery. All participants were given personal access to the webpage. The daily self-practice of mindful walking was reinforced at the end of each class, and self-practice behaviors (i.e., frequency and time spent on self-practice) were queried via telephone and documented on a weekly basis.

**Table 1:** Components of the mHealth-delivered, home-based mindfulness yoga program.

Class	1	2	3	4	5	6	7	8
Theme	Non-judging	Patience	Beginner’s mind	Trust	Non-striving	Acceptance	Letting go	Generosity
Breathing techniques (10 min)	Each class is started by greetings and introduction of each theme, followed by breathing exercises Breathing exercises in easy pose: - Bee breath/ Lion breath - Cooling breath/ Alternate nostril breathing							
Yoga sequence (60 min)	Warm-up sequences: - Easy pose with neck, shoulders, triceps, hands and wrists’ stretch - Easy twist - Cat-cow stretch - Child’s pose Sun salutations sequences, delivered in stepwise approach with modifications (12 poses): - Mountain pose - Upward salute pose - Standing forward bend - Lunge - Plank pose - Knees, chest and chin pose - Cobra pose - Downward-facing dog pose - Lunge - Standing forward bend - Upward salute pose - Mountain pose Cool down sequences: - Child pose - Knee-to-chest - Happy baby - Corpse pose - Side corpse pose and detailed instructions							
Meditation (15 min)	Meditation practice in corpse/easy pose: - Body scan meditation (cultivation of awareness towards breath and body) - Open-monitoring awareness meditation (cultivation of mindfulness towards mind and body) - Loving-kindness meditation (cultivation of loving kindness to oneself and others)							
Sharing and conclusion (5 min)	At the end of each session, the instructor would invite and encourage participants to share their experience, thoughts and feelings of mindfulness yoga practice to promote social interaction, followed by concluding remarks to consolidate learning and reinforce self-practice							
Daily self-practice	Daily 15-min self-practice of mindful walking, guided by pre-recorded video							
Facilitation of self-practice	Guided videos of the same set of yoga sequences, mindful walking, mindfulness meditation, and breathing techniques are accessible on http://yoga.nursing.hku.hk/ (Chinese version) to promote skill mastery and self-practice							

### Online intervention delivery

We invited potential participants to attend a face-to-face individual information session and assessment. Particularly, we reminded them to bring their personal mobile device for rehearsing the online conferencing procedures. An instruction manual summarizing home-based yoga setting and online conferencing procedures was provided with return demonstration. Participants were asked to self-prepare a yoga mat and yoga block. Before the commencement of the online intervention, an individual home-based Zoom session was conducted to ensure an appropriate setting (i.e., a yoga mat with a sturdy chair aside/next to a wall and supportive aids, such as a towel or thick mattress/yoga block). We took specific effort to identify the optimal placement of the smart device and the adjustment of camera’s angle to capture the whole body of participant as much as possible, so that the intervener can respond to the participant’s needs in real-time time. A group-based rehearsal session was also conducted on the day before the first session to ensure a smooth delivery of intervention.

### Safety and adverse events

Safety is particularly important to minimize risks (e.g., fall risks) for patients with PD. All participants were instructed to perform the online yoga practice next to a wall beside a sturdy chair for additional support and with their caregiver(s) nearby if needed. In consideration of the fluctuating motor symptoms and physical functions of patients with PD, two research team members (including an experienced registered nurse in PD care) participated as observers throughout all online yoga sessions to ensure the participants’ safety. If any risk of injury/fall was observed/anticipated, the team would contact the participant via Zoom and/or telephone immediately to reinforce additional safety measures. All participants were instructed to inform the team if they encountered any implementation-related adverse events with medical referrals provided if indicated. Any adverse event would be documented and handled in accordance with the standard operating procedure.

### Data collection and outcome measures

Data regarding sociodemographic characteristics (including age, gender, marital status, education, employment, and income), clinical background (including year of onset, disease staging, and medication record) and the following outcomes were collected at baseline and 1-week post-intervention through Zoom interviews:

### Functional balance

The primary outcome, functional balance was measured with the assessor-rated Berg and Balance Scale (BBS), which has been validated in people with PD [17]. BBS consists of 14 items that assess the balance performance of elderly adults in common everyday life activities on a five-point interval scale (0–4). This scale has a total score that ranges from 0 to 56. A score between 0 and 20 indicates balance deficit, and scores of 21–40 and 41–56 represent acceptable balance and good balance, respectively.

### Severity of motor symptoms

The severity of PD-related motor symptoms was measured with the validated Movement Disorder Society-Unified Parkinson’s Disease Rating Scale (MDS-UPDRS) (Chinese version) part III [18], which measures major PD motor symptoms, including tremors, rigidity, bradykinesia, gait, and postural instability. High scores indicate great disease severity. Motor examination was conducted by qualified personnel with a MDS-UPDRS certificate.

### Perceived balance confidence

Perceived balance confidence was measured with the Activities-specific Balance Confidence (ABC) Scale (Chinese version) [19], which is a validated and reliable tool that is applied to measure the level of self-perceived confidence in balance from 0% (no confidence) to 100% (full confidence) in 16 indoor and outdoor activities. Participants were asked to report their perceived balance confidence during “on” (ABC-on) and “off” (ABC-off) states. A high ABC score indicates a high level of balance confidence.

### Perceived severity of freezing of gait

FOG severity was measured with the validated FOG Questionnaire (Chinese version) (FOGQ) (Cronbach's alpha: 0.94) [20]. FOGQ is a self-reported questionnaire that consists of six items capturing FOG severity and gait. Each item is rated on a five-point interval scale that ranges from 0, which indicates the absence of symptoms, to 4, which indicates the most severe stage of symptoms. The total score of this scale ranges from 0 to 24. A high score indicates severe FOG.

### Anxiety and depressive symptoms

Anxiety and depressive symptoms was measured by applying the validated Hospital Anxiety and Depression Scale (HADS) (Chinese-Cantonese version), which is a self-reported questionnaire that consists of anxiety and depression subscales. Each subscale consists of seven items, and each item is rated on a four-point scale. A high score represents a high level of psychological distress. HADS has been suggested for use in the PD population because somatic symptoms that may potentially overlap with Parkinsonian manifestations are not assessed with this scale [21]. Moreover, it focuses on measuring the negative emotions of anxiety and depression, which have been reported as being the most prominent psychological factors in patients with PD.

### Mindfulness

Mindfulness level was measured with the 20-item validated short-form Five-Facet Mindfulness Questionnaire (Chinese version) [22]. Five key aspects of mindfulness, including observing, describing, acting with awareness, non-judgment of inner experience, and non-reaction to inner experience, were assessed by using a five-point Likert scale. A high score indicates a high level of mindfulness at present.

### HRQOL

HRQOL was assessed by using the validated Parkinson’s Disease Questionnaire-8 (Chinese version) [23, 24]*.* This eight-item scale provides a summary index score that captures disease-specific HRQOL regarding mobility, daily living activities, emotional wellbeing, social support, cognitions, communication, bodily discomfort, and stigma. A high score indicates poor HRQOL.

### Treatment fidelity

For treatment fidelity, exercise intensity in terms of perceived exertion was measured by using the Borg Category-Ratio 10 (CR10) scale at the end of each class. CR10 is a validated tool that is used to quantify an individual’s perception of physical demands during physical work [25, 26]. A CR score ranging from 0 (nothing at all) to 10 (extremely strong) indicates exercise intensity. A score of 4 (somewhat hard) to 7 (very hard) indicates the safe, moderate-to-high intensity of exertion related to the physical workout [27]. Treatment fidelity was assessed by means of virtual ‘on-site’ observation by the first author with the use of a pre-constructed observation checklist.

### Qualitative exploration

Individual semi-structured interviews were conducted to explore the experience of patients with PD in practicing mindfulness yoga through the home-based, mHealth-delivered approach with a focus on conditions that influenced the participants’ motivation, acceptability, real-life practice, and area for improvement. Another area of focus was the participants’ perceived effects of the overall and individual components of the intervention and how and why they work or do not work.

### Statistical analysis

Sociodemographic characteristics and clinical background were summarized using descriptive analyses. Means and SDs were used to describe normally distributed continuous variables, whereas medians and IQRs were used for continuous variables that were not normally distributed. Proportions were used for categorical variables. Pre- and post-intervention comparisons of outcomes between variables at the baseline and the 1-week follow-up were conducted. Paired Student *t*-tests were performed for normally distributed continuous variables, Wilcoxon signed-rank tests were conducted for non-normally distributed variables, and chi-square tests were applied for categorical variables. The analyses were performed by using IBM SPSS version 25.

For qualitative data, audio-taped interviews were transcribed verbatim and managed by using NVivo. Two experienced qualitative researchers inductively analysed the transcribed interviews with a thematic analysis approach [28]. The coded units were sorted into categories and subcategories and analyzed for recurrent themes and patterns. Emerging categories were reviewed for resonance with the quantitative findings. Such qualitative findings served a complementary purpose in reaching conclusions on the preliminary effects and acceptability of home-based, mHealth-delivered mindfulness yoga practice for PD rehabilitation.

## Results

### Patient engagement

**Fig. 1:**
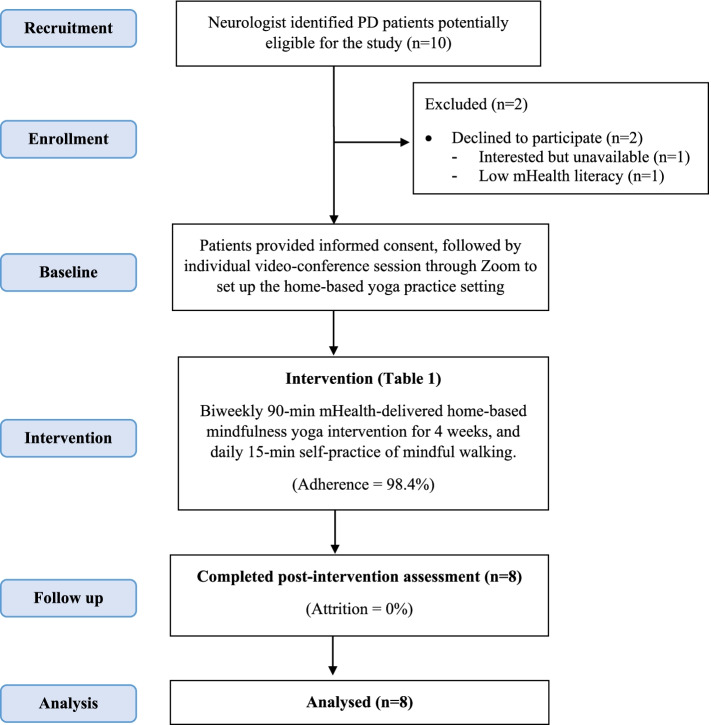
CONSORT 2010 flow diagram

**Fig. 2:**
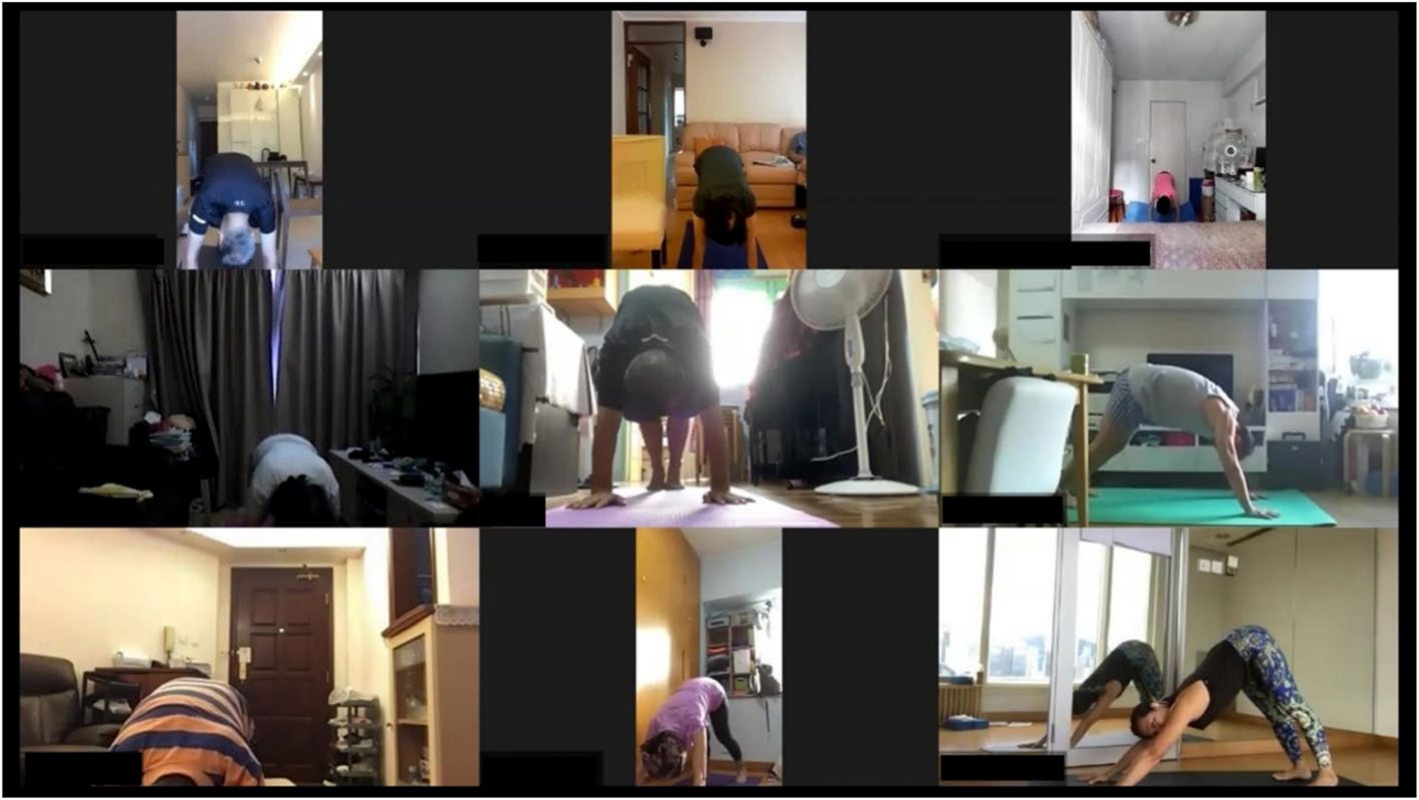
Screen capture of the intervention session (8 participants and 1 instructor, consent obtained)

Figure 1 depicts the study flow. With the COVID-19 outbreak, all out-patient services were kept minimal. Among the ten potential participants screened, all met the eligibility criteria and one declined to participate (enrolment rate: 80%) due to unavailability and another declined because of mHealth illiteracy. Figure 2 shows the screen capture of one intervention session. The mean and standard deviation (SD) attendance rates were 7.88 (0.33) sessions with a participation rate of 98.4%. One participant did not attend one session due to a schedule conflict with a medical appointment. All participants attended the online follow-up assessment sessions. The compliance rates for the daily 15-min mindful walking self-practice during the intervention period were 100%. The mean (SD) frequencies of mindful walking were 1.8 (0.2) times per day. No serious adverse event was observed in any of the participants.

### Patients’ characteristics

The participants’ sociodemographic and clinical characteristics are summarized in Table 2. The mean age of participants was 63.1 years (SD 5.4 years), and half of the participants were male. The majority of the patients were married (62.5%) and received tertiary education (62.5%). All were living with a spouse/family, and half received social security allowance. All had moderate PD (Hoehn and Yahr scale, stage 3). Their mean (SD) levodopa equivalent dose was 738.4 (469.2).

**Table 2:** Baseline sociodemographic and clinical characteristics of the participants

Characteristics	Participants (*n* = 8), mean (SD) or no. (%)
Age	63.1 (5.4)
Gender	
Male	4 (50.0)
Female	4 (50.0)
Marital Status	
Single, widowed	3 (37.5)
Married	5 (62.5)
No. of children	1.6 (0.7)
Educational level	
Secondary	3 (37.5)
Tertiary	5 (62.5)
Living with spouse or family	8 (100)
Social security allowance	4 (50)
Hoehn and Yahr stage	3 (0)
Levodopa equivalent dose	738.38 (469.2)

### Tele-assessment of outcome measures

All outcomes were assessed uneventfully using Zoom. Participants were familiar with videoconferencing procedures and followed commands of physical examination. Table 3 presents the scores of all outcome variables at baseline and 1-week follow-up.

**Table 3:** Results of the outcome variables (*n* = 8)

**Outcome**	**T0, Mean (SD)**	**T1, Mean (SD)**	**Change (95% CI)**	***P* Value**
ABC-ON §	87.07 (10.56)	85.30 (10.27)	3.63 (-3.73 to 11.00)	.28
ABC-OFF§	61.72 (13.55)	74.94 (18.64)	-13.22 (-32.09 to 5.65)	.14
BBS §	50.00 (2.20)	55.13 (1.73)	-5.13 (-6.61 to -3.61)	< .001
MDS-UPDRS III*	25.25 (5.90)	12.63 (4.50)	12.63 (6.52 to 18.73)	.002
FOGQ	12.38 (5.04)	11.00 (4.24)	1.38 (-1.11 to 3.86)	.23
HADS-anxiety	12.75 (2.77)	3.75 (2.82)	9.00 (4.64 to 13.36)	.002
HADS-depression	8.88 (2.03)	5.25 (3.73)	3.63 (0.31 to 6.94)	.04
FFMQ-Total				.82
FFMQ-Observing	14.13 (4.26)	13.38 (3.66)	0.75 (-1.43 to 2.93)	.44
FFMQ-Describing	16.50 (2.14)	14.13 (3.18)	2.38 (-1.17 to 5.92)	.16
FFMQ-Acting with awareness	14.37 (3.62)	15.13 (2.23)	-0.75 (-3.46 to 1.96)	.53
FFMQ-Non-judgment	10.13 (3.60)	12.38 (2.00)	-2.25 (-4.56 to 0.06)	.055
FFMQ-Non-reaction	15.38 (2.13)	14.75 (1.39)	0.63 (-1.47 to 2.72)	.50
PDQ-8 summary index	24.22 (14.05)	23.44 (11.93)	0.78 (-6.96 to 8.53)	.82

Abbreviations ABC, Activities-Specific Balance Confidence Scale percentage confidence; on, on medication state; off, off medication state, BBS, Berg Balance Scale, FFMQ, Five Facet Mindfulness Questionnaire; FOGQ, Freezing of Gait Questionnaire, HADS, Hospital Anxiety and Depression Scale, MDS-UPDRS III, Movement Disorders Society United Parkinson’s Disease Rating Scale, Part III (excluded item 3.3 and 3.12 due to virtual assessment), PDQ-8, 8-item Parkinson’s Disease Questionnaire. All assessments were conducted virtually via Zoom at baseline (T0) and immediately post-intervention (T1). ^§^an negative change score indicates improvement

### Physical outcomes

Participants showed a significant improvement in functional balance (BBS: mean change = 5.13; *p* < 0.001; Cohen’s d = 2.84) and overall motor symptoms (MDS-UPDRS-III: mean change = 12.63; *P* = 0.002; Cohen's d = 2.33) from baseline to 1-week follow-up. For subjective measures, perceived balance confidence during the “on” (ABC-ON: mean change = 3.63; *P* = 0.28) and “off” states (ABC-OFF: mean change = -13.22; *P* = 0.14) and perceived FOG (FOGQ: mean change = 1.38; *P* = 0.23) were insignificant.

### Mental health and HRQOL outcomes

For psychological distress, participants showed a significant reduction in anxiety (HADS-anxiety: mean change = 9.00; *P* = 0.002; Cohen's d = 1.73) and depressive symptoms (HADS-depression: mean change = 3.63; *P* = 0.04; Cohen's d = 0.91). For mindfulness, an improving trend was noted regarding the domain of “non-judging to inner experience” (FFMQ-non-judging: mean change = -2.25; *P* = 0.05). HRQOL showed insignificant improvement (PDQ-8: mean change = 0.78; *P* = 0.82).

### Feasibility, treatment intensity and safety of the mindfulness yoga programme

We recruited 10 eligible participants from the neurology out-patient clinic, with an enrolment rate of 80%. The adherence rate of online intervention was 98.4%, in which one participant missed a class due to a schedule clash with medical appointment. The mean (SD) of the Borg CR10 was 5.68 (1.03) with a range of 4 to 8, indicating the safety and moderate intensity of the exercise. All online sessions demonstrated a satisfactory treatment fidelity. The self-practice of mindful walking had a mean (SD) time of 23.13 (12.64) minutes per day and ranged from 10 to 60 min per day. All participants attended the pre- and post-intervention follow-ups. For safety concerns, one participant exhibited instability due to dyskinesia during the first virtual class on yoga practice. Our team immediately contacted the participant through Zoom and reinforced safety precautions, including practicing next to a wall and placing a chair next to the yoga mat for support. Overall, all classes were delivered uneventfully without an adverse event.

### Qualitative feedback

Participants reported that they had positive experience with the intervention. They identified several merits of the intervention as follows:

The mindfulness yoga program was interactive and interesting, thus greatly increasing their motivation in establishing sustained practice. The participants requested additional online sessions and verbalized remarkable commitment and interest in attending long-term mHealth-delivered home-based mindfulness yoga for rehabilitation.

“*I did not expect exercising at home could be so enjoyable. It was really fun to practice in a group virtually, and the interactive real-time approach using Zoom really kept us motivated. I want to have more of it.*” (Participant 2)

The home-based approach was convenient and reassuring, which reduced the burden associated with transportation.

*“I used to spend over an hour in travelling to attend supervised exercise classes. Now, it’s convenient that we can practice at home. I can easily integrate it (the program) into my daily routine without spending extra time and money on transportation.”*(Participant 1)

Moreover, the participants’ adherence would not be hindered by common barriers to participation in center-based programs, such as adverse weather, the recent public health measures against COVID-19, and safety concerns related to social movement activities in the local community.

*“I worried about going out since the start of the social movements, and it’s now even worse due to the COVID-19 outbreak. I felt like being trapped at home and I was much stiffer. It’s so great that I could be active again (by joining this program) even at home.”*(Participant 5)

The participants also commented that the home-based yoga setup via Zoom was manageable because it required only a yoga mat, chair, and one smart device installed with Zoom. The assistance from caregiver/families is deemed helpful to facilitate the set up.

*“Although I am not very familiar with computer and Zoom, it’s quite easy with the demonstration and return demonstration (by the research team), and my husband helped me with that.”* (Participant 4)

They verbalized concerns about the execution of yoga postures and suggested that ideally, one to two fundamental in-person yoga classes should be provided before virtual classes to enhance skill mastery.

*“Although the instructor taught step-by-step and I can follow the yoga sequences, I am not sure if I followed the steps correctly. It would be ideal if there’s one to two in-person classes beforehand, but I understand that it might not be feasible during the COVID-19.”* (Participant 3)

The overall comments on the intervention effects covered a range of physical and mental benefits including improved mood and sleep and enhanced calmness, balance, mobility, and gait. Regarding intervention components, all participants reported that the mindful walking practice was associated with improved walking confidence, improved gait control during gait initiation and walking, and reduced FOG episodes. Such benefits were particularly apparent during the “off” state.

*“During the mindful walking, I felt like I learned how to walk again, and my mind was reconnected to my legs and feet. The overall program really helped my body coordination, especially during my off-time, I was less anxious and could function better.”* (Participant 5)

The participants felt calm and relaxed after practicing breathing exercises, and almost 40% reported that meditation practices were associated with improved sleeping quality and emotional stability.

## Discussion

To the best of our knowledge, this prospective pilot study is the first attempt to test the feasibility of a home-based interactive mindfulness yoga intervention through an online platform to help promote the physio–psycho–spiritual well-being of patients with PD. The findings of this study indicated that the mHealth-delivered home-based mindfulness yoga programme was feasible and safe without adverse effects, and particularly well received for patients with PD during the COVID-19 pandemic.

For people with PD, adhering to rehabilitation programs is particularly crucial and becomes challenging as they experience progressive motor dysfunctions and mental complications over the course of their illness. Patients with PD begin to experience motor fluctuations, including wearing-off effects and ‘on–off’ phenomena, with increasing frequency after 2–5 years on average due to the short and/or unpredictable response time of long-term levodopa use [29]. All these complications considerably affect their functional performance, planned schedule, motivation, and adherence to participation in center-based programs. A 2016 systematic review of mind–body exercises reported that an increase in the intervention frequency may be associated with unfavorable enrolment and adherence among people with PD [5]. Previous studies on center-based mind–body exercise interventions with a weekly frequency reported that the adherence rates of participants with PD range from 56.4% to 92.9% [7, 30–32]. Our pilot findings demonstrated that the biweekly mindfulness yoga program was remarkably well-received among patients with PD despite its increased frequency as evidenced by its high enrolment and adherence rates of 80% and 98.4%, respectively. This finding highlighted the particular relevance and importance of the mHealth-delivered home-based approach to PD rehabilitation. Such an approach has low resource demands and removes the common external barriers to participation in center-based programs [33]. These barriers include adverse weather events, the social distancing and public health measures used to combat the COVID-19 pandemic, and safety concerns related to social movement activities in the local community. In addition, delivering yoga in a familiar and controlled home setting may facilitate the patients’ adherence and sustained engagement in frequent yoga practice.

Previous evidence showed that the high frequency of yoga practice is associated with low emotional and physiological reactivity, high emotional stability, and resilience to negative stimuli or vulnerability [34, 35], as well as increased physical health benefits among older adults [36]. More frequent yoga practice could deepen one’s mind–body awareness and connections [37]. However, its effects on cultivating mindfulness attitudes remained unknown. The virtual delivery of yoga practice may affect the immersion of participants in “in-moment” experiences and may compromise the quality of present-moment awareness and thus the cultivation of mindfulness. As such, the intervention effect may be mediated through the psychosocial effects of virtual gatherings or the physiological effects of exercise rather than the cultivation of mindfulness attitudes. Hence, future trials should adopt an active control group for comparison and investigate the mediating roles of physical performance and mindfulness level in the effect of mindfulness training on psychological distress and HRQOL, as well as strategies optimizing the “in-moment” experiences of virtually delivered mindfulness yoga practice. Mocanu and Mohr [34] found that long-term yoga practice is linked with high “moment-to-moment” consciousness and considerable improvement in clinical symptoms and life satisfaction. Hence, we suggest prolonging the duration of future mind–body interventions to achieve subjective improvement. In corroboration with previous studies on mind–body exercises for PD [5, 7], delivering interventions through biweekly dosages for 8–10 weeks is essential to enhance the skill mastery of mindfulness yoga and promote physio–psycho–spiritual gains, as well as ensure adequate power and time to translate objective functional improvements into subjective positive experience that can be perceived by patients in daily living.

Corroborating with a recent report of PD management during the COVID-19 pandemic [38], the findings of the present study also supported the implementation of telemedicine to assess physical symptoms of PD. Indeed, previous studies [39, 40] have demonstrated that most physical symptoms can be visualized and many core features of PD, except rigidity and postural reflexes, can be captured and rated through videoconference. This pilot study has further demonstrated the feasibility of tele-assessment of physical symptoms in local setting, given the fact that a stable bandwidth is essential to ensure an optimal video quality in terms of resolution and smoothness of motion. Although there is some loss of information when using tele-approach to rate BBS/MDS-UPDRS-III compared to in-person ratings, such tele-assessment approach may serve as a low-cost and convenient alternative to facilitate the continuum of PD care in the community.

Last but not least, patient qualitative data indicated that mindful walking was particularly helpful in improving gait control and FOG symptoms, particularly during wearing-off. The high compliance of mindful walking self-practice suggests participants’ high acceptance to this complementary meditative practice, which can be practiced anywhere without any equipment or restrictions. Indeed, managing FOG is difficult owing to its multifaceted pathophysiology, which involves an interplay between motor elements (dysregulated stepping mechanisms) and non-motor elements (anxiety and cognitive decline) [2]. Most research to date has examined the effects of center-based motor skill training combined with external cueing strategies (i.e., rhythmic auditor stimulation) for FOG management [41, 42]. However, such modalities cannot be implemented in the form of ambulatory strategies to assist patients in their daily activities regardless of their location. The self-practice and internalizing nature of mindful walking techniques can potentially be adopted as an ambulatory strategy that can readily be integrated into daily living to enhance body coordination and alleviate FOG symptoms. Mindfulness techniques may serve as an internal cueing strategy for mental activation to enhance “in-present” mind–body awareness and coordination, which present promising therapeutic potential for PD rehabilitation. The incorporation of mindfulness and motor skill training may provide additional avenues by which a person can improve mind–body coordination for FOG management. Its potential clinical values should be further evaluated by using a methodological design with increased rigor, i.e., a randomized controlled design with a large sample size.

### Implications

The home-based mindfulness yoga intervention, which was delivered interactively via an online platform and utilized prerecorded videos for self-practice, gave a unique opportunity to provide much-needed mind–body rehabilitation to patients with PD for the likely improvement of functional balance, motor symptoms, and mental status. This feasibility study showed that the mHealth-delivered home-based approach is a feasible, safe, and well-received option for patients with PD, in particularly during the COVID-19 pandemic. The experience of this study might aid the development of large randomized controlled trials or similar programs for patients with PD or other neurological conditions, especially those with physical impairment or limited resources who are unable to reach center-based rehabilitation programs frequently. Moreover, it appeared to be particularly helpful and accessible during times of infectious disease outbreaks. We recommend increasing the intervention training sessions to twice a week for 8–10 weeks to enhance treatment effects, especially effects on the component of subjective functional outcomes and HRQOL. The effects of mindful walking should be further evaluated in future trials for FOG management.

### Limitations

Despite its positive outcomes, this study had several limitations. First, the sample size was small and a comparison group was not established; thus, caution is needed when interpreting its findings. Second, some features of PD, including rigidity and postural reflexes, cannot be captured and assessed on videoconference. Third, adherence to mindful walking was self-reported and thus might be overestimated owing to social desirability bias and recall bias. Future studies should adopt a large sample size and preferably use a randomized controlled trial design. The subjective experience of the participants should also be explored by collecting qualitative feedback throughout the study, and the sustained effects of the intervention should be examined with a lengthy follow-up.

## Conclusions

This study provided evidence supporting the feasibility of the mHealth-delivered home-based mindfulness yoga program to people with mild-to-moderate PD. Although motor dysfunctions and PD pathology have been identified as factors that might challenge the feasibility, safety, and acceptability of virtual rehabilitation in this vulnerable group, this pilot study showed that the mHealth-delivered home-based mindfulness yoga intervention was feasible, safe and well-accepted among people with PD, which was particularly helpful and accessible during times of infectious disease outbreaks. Future studies with enhanced rigor and increased sample size are needed to establish efficacy in patients with PD and those with other neurological conditions or physical impairments.

## Data Availability

The datasets generated and/or analysed during the current study are not publicly available but are available from the corresponding author on reasonable request.
